# Exploring the Relationship Between Visceral Fat and Coronary Artery Calcification Risk Using Metabolic Score for Visceral Fat (METS-VF)

**DOI:** 10.3390/life14111399

**Published:** 2024-10-31

**Authors:** Jiun-Chi Huang, Ya-Chin Huang, Chia-Hsin Lu, Yun-Shiuan Chuang, Hsu-Han Chien, Chia-I Lin, Ming-Fang Chao, Hung-Yi Chuang, Chi-Kung Ho, Chao-Ling Wang, Chia-Yen Dai

**Affiliations:** 1 Department of Internal Medicine, Kaohsiung Municipal Siaogang Hospital, Kaohsiung Medical University, Kaohsiung 812, Taiwan; karajan77@gmail.com; 2 Division of Nephrology, Department of Internal Medicine, Kaohsiung Medical University Hospital, Kaohsiung Medical University, Kaohsiung 807, Taiwan; 3 Research Center for Precision Environmental Medicine, Kaohsiung Medical University, Kaohsiung 807, Taiwan; 4 Department of Preventive Medicine, Kaohsiung Municipal Ta-Tung Hospital, Kaohsiung Medical University, Kaohsiung 801, Taiwan; jasimine0603@gmail.com (Y.-C.H.); yik89045@gmail.com (H.-H.C.); linchiai@gmail.com (C.-I.L.); 5 Department of Occupational & Environmental Medicine, Kaohsiung Medical University Hospital, Kaohsiung Medical University, Kaohsiung 807, Taiwan; hychuang@gmail.com (H.-Y.C.); hochikung@yahoo.com (C.-K.H.); 6 Department of Medical Imaging, Kaohsiung Municipal Ta-Tung Hospital, Kaohsiung Medical University Hospital, Kaohsiung Medical University, Kaohsiung 807, Taiwan; freebone46@yahoo.com.tw; 7 Department of Family Medicine, Kaohsiung Medical University Hospital, Kaohsiung Medical University, Kaohsiung 807, Taiwan; kinkipag@gmail.com; 8 Department of Medical Imaging, Kaohsiung Medical University Hospital, Kaohsiung Medical University, Kaohsiung 807, Taiwan; chaominfang@gmail.com; 9 Hepatobiliary Division, Department of Internal Medicine, Kaohsiung Medical University Hospital, Kaohsiung Medical University, Kaohsiung 807, Taiwan

**Keywords:** cardiovascular disease, METS-VF, obesity, coronary artery calcium score, anthropometric index, visceral fat

## Abstract

Background: Metabolic Score for Visceral Fat (METS-VF) is a novel indicator for estimating intra-abdominal fat, yet its connection with coronary artery calcification (CAC) remains uncharted. Our research aims to explore the novel METS-VF indicator’s link to CAC while comparing its performance against relevant anthropometric indices. Methods: This study enrolled participants who underwent health checkups and computed tomography scans for categorizing severity of CAC using the coronary artery calcium score. The METS-VF was calculated and compared with anthropometric indices in estimating the presence of CAC and different CAC severity using receiver operating characteristic curves. Results: Overall, 1217 participants (mean age 50.7 ± 9.9, 53.8% male) were included. METS-VF (odds ratio [OR], 1.506; 95% confidence interval [CI], 1.181–1.921; *p* = 0.001) was positively associated with the presence of CAC, even after accounting for cardiometabolic factors. Notably, METS-VF was positively associated with mild (OR, 1.450; 95% CI, 1.115–1.886; *p* = 0.006), moderate (OR, 1.865; 95% CI, 1.137–3.062; *p* = 0.014), and severe (OR, 2.316; 95% CI, 1.090–4.923; *p* = 0.029) CAC. Moreover, METS-VF yielded the highest area under curve (AUC) value in the estimation of the CAC presence (AUC = 0.710), mild (AUC = 0.682), moderate (AUC = 0.757), and severe (AUC = 0.807) CAC when compared with body mass index, waist circumference, visceral adiposity index, triglyceride–glucose index, and metabolic score for insulin resistance. The optimal METS-VF cut-off value was 6.4 for predicting CAC. Conclusions: METS-VF emerged as a strong independent marker for detecting CAC presence across mild, moderate, and severe CAC categories, outperforming major anthropometric indices in accurately estimating the presence of CAC and different severity of CAC.

## 1. Introduction

Currently, cardiovascular disease (CVD) remains the foremost contributor to mortality and imposes a significant economic burden globally [1]. Several risk factors for CVD, such as hypertension, dyslipidemia, and dietary factors are modifiable. However, nearly half of all CVD-related deaths have no previous CVD diagnoses, highlighting the crucial need for proper risk stratification in asymptomatic individuals [2]. To estimate the atherosclerosis burden, the coronary artery calcium score (CACS) is calculated through computed tomography (CT) scans. Agatston score is a well-established method for the quantification of the CACS, which is used as a reference for most population databases and publications involving risk stratification and is often used in clinical practice [3]. An elevated CACS is closely linked to heightened cardiovascular event risks and all-cause mortality [4]. Notably, the Multi-Ethnic Study of Atherosclerosis has underscored CACS as the most robust prognostic indicator for coronary heart disease, surpassing novel biomarkers and traditional risk factors [5]. In a practical setting, CACS is utilized for CVD risk discussions, facilitating clinical decision-making, setting treatment goals, and considering cost-effectiveness [6].

Visceral fat presents as an independent risk for CAC, type 2 diabetes, CVD, and all-cause mortality [7,8,9]. While bioelectrical impedance analysis and dual-energy X-ray absorptiometry are commonly used methods for assessing body composition, their accuracy is limited in providing specific information about adipose tissue distribution [10]. Ultrasonography, another prevalent method, assesses superficial adipose tissue and epicardial adipose tissue thickness, but it is operator-dependent and lacks comprehensive insight into most visceral adipose tissue (VAT) deposits. CT and magnetic resonance imaging offer accurate measurements of visceral fat but are primarily used in research due to high costs and equipment requirements [11]. The recently developed Metabolic Score for Visceral Fat (METS-VF) introduces a novel approach for estimating visceral fat. It has been reported that METS-VF shows a stronger correlation and better diagnostic performance compared to other VAT surrogates, such as body mass index (BMI), waist circumference (WC), waist-to-height ratio, and the visceral adiposity index (VAI) [12]. Comprising components of insulin resistance, anthropometric measures of body-fat distribution, age, and sex, MET-VF exhibits robust predictive capabilities in detecting metabolic syndrome [12], anticipating type 2 diabetes and hypertension development [13,14], and assessing the risk of chronic kidney disease [15].

The connection between anthropometric indices and CACS is well-established, particularly the correlation between obesity and elevated CAC risk [16]. WC has demonstrated greater predictability for CACS compared to BMI in certain populations [17]. Furthermore, the VAI, reflecting higher visceral adiposity, is significantly associated with elevated CAC risk [18]. The triglyceride–glucose (TyG) index, a reliable surrogate for insulin resistance, has been independently linked to CACS and progression [19]. A metabolic score for insulin resistance (METS-IR) combines non-insulin fasting laboratory values and anthropometric measurements to assess insulin resistance and is valuable for evaluating cardiac metabolic risk [20]. METS-VF is a novel indicator for estimating visceral fat, but its relationship with CAC has not been clearly investigated. The primary objective of this study is to investigate the relationship between METS-VF and the presence as well as the different severity of CAC. Secondly, we aimed to compare the performance of METS-VF with METS-IR and major anthropometric indices, including BMI, WC, VAI, and the TyG index, in estimating the presence and different severity of CAC.

## 2. Materials and Methods

### 2.1. Study Design and Participants

**Figure 1:**
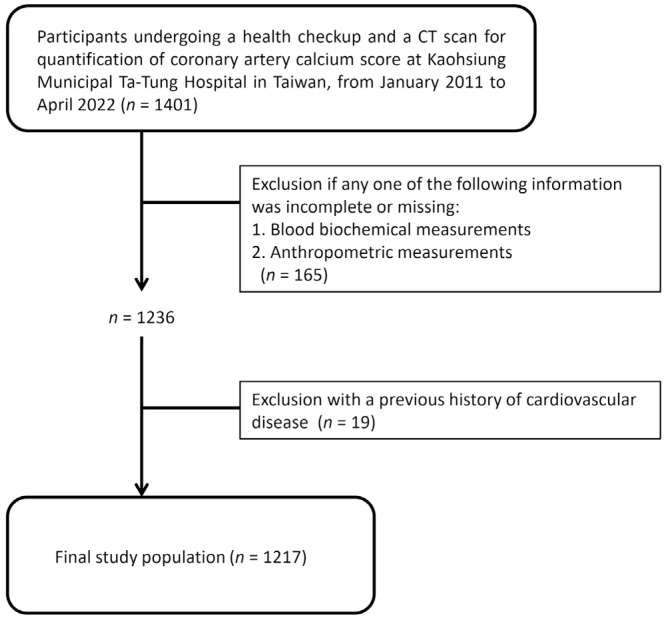
Study flowchart.

This study enrolled 1401 participants who received a health checkup and underwent a CT scan for quantification of CACS at Kaohsiung Municipal Ta-Tung Hospital in Taiwan from January 2011 to April 2022. To ensure data integrity, 165 participants with incomplete blood biochemical or anthropometric measurements were excluded from the analysis. In addition, 19 participants with previous history of CVD were also excluded. Overall, 1217 participants were included in this study (Figure 1). Face-to-face interviews were conducted with all participants, during which physicians evaluated their medical history and collected relevant information, including age, sex, and history of CVD. Smoking status was categorized as a current smoker or not. Additionally, anthropometric measurements, including height and weight, were measured by well-trained staff. BMI was defined as body weight in kilograms divided by the square of height in meters. WC was measured by placing a non-stretchable tape around the midpoint between the highest point of the iliac crest and the lowest rib. Blood pressure was measured after at least five minutes of seated rest. This study was conducted in accordance with the Declaration of Helsinki, and the study protocol was approved by the Institutional Review Board (IRB) of Kaohsiung Medical University Hospital (KMUHIRB-E(I)-20220120 approved on 30 June 2022).

### 2.2. Laboratory Examinations

Biochemical measurements were performed using at least 10 h fasting blood samples from the participants. An automated analyzer (TBA-c16000, Toshiba, Tokyo, Japan) was utilized to measure several parameters including fasting glucose (reference values 70–100 mg/dL), total cholesterol (reference values < 200 mg/dL), triglyceride (TG) (reference values < 150 mg/dL), high-density lipoprotein (HDL) cholesterol (reference values > 40 mg/dL for adult males, >50 mg/dL for adult females), low-density lipoprotein (LDL) cholesterol (reference values < 130 mg/dL), uric acid (reference values 3.5–7.2 mg/dL for adult males, 2.6–6.0 mg/dL for adult females), and serum creatinine (reference values 0.72–1.25 mg/dL for adult males, 0.57–1.11 mg/dL for adult females) levels. The estimated glomerular filtration rate (eGFR) was assessed using the Chronic Kidney Disease Epidemiology Collaboration equation [21], a recommended method for assessing kidney function in adults.

### 2.3. Definitions of Hypertension and Diabetes

Participants’ blood pressure (BP) levels were measured using an electronic, automatic device in an undisturbed and quiet setting, with a rest period at least 5 min prior to the measurement. BP was recorded three times at 1–2 min intervals, and the average of these measurements was used for analysis. Hypertension was defined as a systolic blood pressure of ≥140 mmHg and/or diastolic blood pressure of ≥90 mmHg, or self-reported use of antihypertensive medication [22]. Diabetes was defined as a fasting glucose level of ≥126 mg/dL or self-reported use of insulin or antidiabetic agents [23].

### 2.4. Definitions of METS-VF, METS-IR, VAI, and TyG Index

The METS-VF was calculated using the following formula: 4.466 + 0.011 × (Ln(METS-IR))^3^ + 3.239 × (Ln(Waist-to-height index))^3^ + 0.319 × (Sex) + 0.594 × (Ln(Age)) [12]. The METS-IR was determined as Ln((2 × G_0_) + TG_0_) × BMI)/(Ln(HDL cholesterol)) where G_0_ and TG_0_ represent fasting glucose and TG concentrations, respectively [20].

The formulas used to calculate VAI and TyG index were listed as follows [19,24]: VAI for men = WC (cm)/(39.68 + (1.88 × BMI)) × (TG (mmol/L)/1.03) × (1.31/HDL cholesterol (mmol/L)); VAI for women = WC (cm)/(36.58 + (1.89 × BMI)) × (TG (mmol/L)/0.81) × (1.52/HDL cholesterol (mmol/L)); TyG Index = Ln(fasting TG × fasting glucose/2).

### 2.5. CACS Measurements and Severity of CAC

All participants underwent CACS measurements by a 640-slice multidetector CT scanner (Aquilion ONE, Toshiba, Tokyo, Japan) with the Calcium Score eXam Plan. A prospective ECG-gating protocol utilizing a step-and-shoot technique was employed for the imaging procedure. All participants were positioned in the supine position and instructed to hold their breath during the imaging process. The analysis of coronary CT images was performed by well-trained radiologists. The CACS was automatically quantified using dedicated software, and the severity was evaluated based on the Agatston score [3]. Based on the CACS obtained, the presence of CAC was defined as a CACS > 0. Additionally, the severity of the CAC was classified into four categories: zero (CACS = 0), mild (CACS 1 to 99), moderate (CACS 100 to 399), and severe (CACS ≥ 400) [25].

### 2.6. Statistical Analysis

The study subjects were categorized into three groups based on the tertiles of their METS-VF levels. Categorical variables were expressed as numbers and percentages, while continuous variables were presented as means ± standard deviation or medians (25th–75th percentile). The Chi-square test was used to analyze categorical variables, and one-way analysis of variance was employed to compare means for continuous variables. Spearman’s correlation was utilized to assess the relationship of METS-VF and anthropometric indices with CACS. Non-adjusted and adjusted binomial logistic regression analyses were conducted to assess the association of METS-VF and anthropometric indices (standardized by Z scores) with the presence of CAC. Adjustments were made for potential confounders, including age, sex, hypertension, diabetes, current smoking status, total cholesterol, HDL cholesterol, LDL cholesterol, TG, uric acid, and eGFR. Additionally, the association among METS-VF, major anthropometric indices, and different severity of CAC were identified by logistic regression analyses after adjusting main cardiovascular risk factors. Receiver operating characteristic (ROC) curve analysis and the area under the curve (AUC) were used to assess the performance of METS-VF and anthropometric indices in predicting the presence of CAC (CACS > 0), mild CAC (CACS 1–99), moderate CAC (CACS 100–399), and severe CAC (CACS ≥ 400). The optimal cut-off points of METS-VF for prediction of the presence of CAC, mild CAC, moderate CAC, and severe CAC were determined by maximizing the Youden index. DeLong’s test was used to compare significant differences in AUC among these markers. The integrated discrimination improvement (IDI) and net reclassification improvement (NRI) were also assessed to quantify the improvement in predictive ability by adding METS-VF. Statistical analyses were performed using SAS version 9.4 (SAS Institute, Cary, NC, USA) and SPSS version 22.0 (IBM Corp., Armonk, NY, USA). A two-tailed *p*-value < 0.05 was considered statistically significant.

## 3. Results

### 3.1. Characteristics of Study Participants Stratified by Tertiles of METS-VF

A total of 1217 participants were included and stratified according to the tertiles of METS-VF in this study. The biochemical parameters and clinical characteristics of the study subjects are presented in Table 1. The participants had a mean age of 50.7 ± 9.9 years, with 53.8% of them being male, and 375 (30.8%) individuals had a CACS > 0. Participants in the group of the highest METS-VF tertile were more likely to have an older age, higher blood pressure, higher proportion of males, hypertension, diabetes, and current smoking, higher levels of fasting glucose, uric acid, TG, serum creatinine, and lower levels of total cholesterol, HDL cholesterol, and eGFR. Furthermore, anthropometric indices, including WC, BMI, VAI, TyG index, and METS-IR, also exhibited significant differences among the groups. Importantly, the proportion of CACS > 0 and the severity of CAC significantly increased with an increase in METS-VF. As shown in Figure S1, the value of METS-VF increased with the increase in severity of CAC (*p* for trend < 0.001).

### 3.2. Associations of METS-VF and Anthropometric Indices with the Presence of CAC

Spearman’s correlation analysis revealed positive correlations between METS-VF, anthropometric measurements, and CACS, with METS-VF showing the strongest correlation coefficient (Table 2).

**Figure 2:**
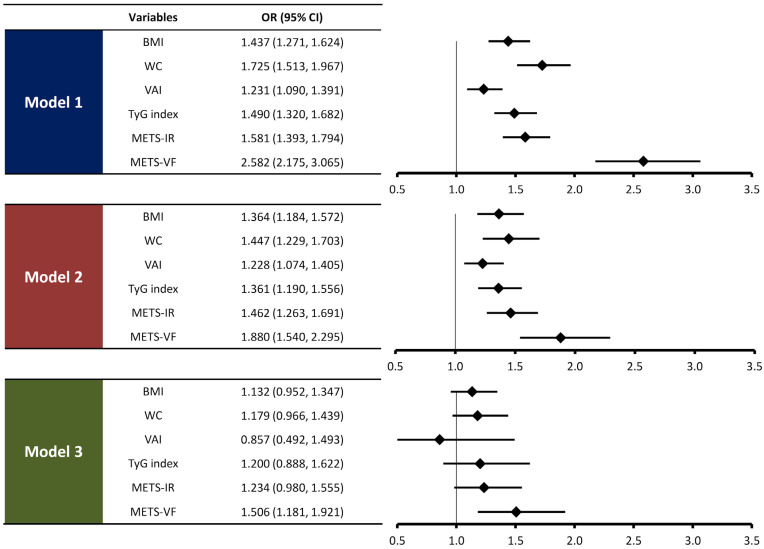
Associations of METS-VF and anthropometric indices with the presence of CAC. The ORs were expressed per 1-standard deviation change in METS-VF and other anthropometric indices. Model 1: no adjustment. Model 2: adjusted for age and sex. Model 3: model 2 plus hypertension, diabetes, current smoking, total cholesterol, HDL cholesterol, LDL cholesterol, triglyceride, uric acid, and eGFR.

As shown in Figure 2, BMI, WC, VAI, TyG index, METS-IR, and METS-VF were associated with the presence of CAC in unadjusted logistic regression analysis. After adjusting for age and sex, METS-VF (odds ratio [OR], 1.880; 95% confidence interval [CI], 1.540–2.295; *p* < 0.001) demonstrated a significant association with the presence of CAC. Following further adjustment of main cardiometabolic risk factors in addition to age and sex, METS-VF (OR, 1.506; 95% CI, 1.181–1.921; *p* = 0.001) was significantly associated with the presence of CAC. However, the association with the presence of CAC was not observed in BMI, WC, VAI, TyG index, and METS-IR in multivariate logistic regression analysis.

### 3.3. Associations of METS-VF and Anthropometric Indices with Mild, Moderate, and Severe CAC

We further examined the associations of METS-VF and major anthropometric indices with different categories of CAC severity, as shown in Table 3. In univariate logistic regression analysis, METS-VF and all major anthropometric indices, including BMI, WC, VAI, TyG index, and METS-IR, were associated with mild, moderate, and severe CAC, respectively. In multivariate logistic regression analysis, METS-VF remained significantly associated with mild CAC (OR, 1.450; 95% CI, 1.115–1.886; *p* = 0.006), moderate CAC (OR, 1.865; 95% CI, 1.137–3.062; *p* = 0.014), and severe CAC (OR, 2.316; 95% CI, 1.090–4.923; *p* = 0.029) after adjusting for age, sex, and main cardiometabolic risk factors. The associations between major anthropometric indices with different categories of CAC severity were not observed in the adjusted model of analysis, except for the association of the TyG index (OR, 2.069; 95% CI, 1.122–3.815; *p* = 0.020) with moderate CAC.

### 3.4. Performance of METS-VF and Anthropometric Indices in Estimating the Presence of CAC, Mild CAC, Moderate CAC, and Severe CAC

**Figure 3:**
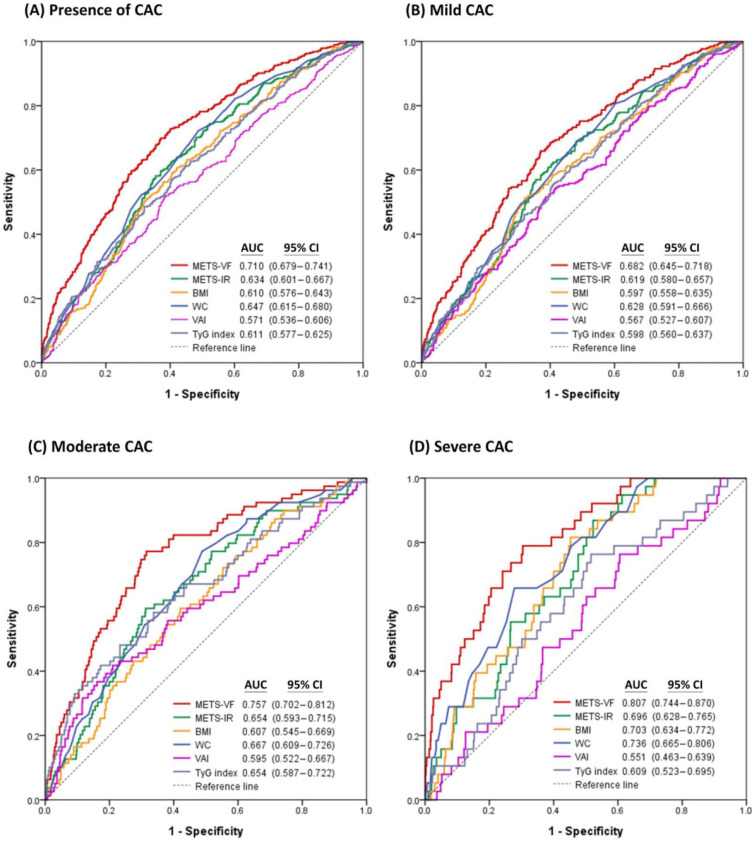
Receiver operating characteristic curves of METS-VF and major anthropometric indices for estimating (**A**) the presence of CAC, (**B**) mild CAC, (**C**) moderate CAC, and (**D**) severe CAC.

Figure 3 demonstrates the comparisons of performance in estimating the presence of CAC and different categories of CAC severity among METS-VF and major anthropometric indices. Notably, METS-VF exhibited the greatest AUC value for estimating the presence of CAC (AUC = 0.710; 95% CI, 0.679–0.741), mild CAC (AUC = 0.682; 95% CI, 0.645–0.718), moderate CAC (AUC = 0.757; 95% CI, 0.702–0.812), and severe CAC (AUC = 0.807; 95% CI, 0.744–0.870) among the major anthropometric indices. When comparing the AUC of METS-VF as a reference, the differences in AUC between METS-VF and each anthropometric index were all statistically significant using DeLong’s test for the presence of CAC (all *p* < 0.001), mild CAC (all *p* < 0.001), moderate CAC (all *p* < 0.01), and severe CAC (all *p* < 0.001) (Table S1).

The predictive model for the presence of CAC was significantly enhanced by including METS-VF, even after adjusting for age, sex, hypertension, diabetes, current smoking, total cholesterol, HDL cholesterol, LDL cholesterol, triglyceride, uric acid, and eGFR (Table S2). Additionally, incorporating METS-VF into the model significantly improved the NRI and IDI compared to models that included BMI, WC, VAI, TyG index, or METS-IR (Table S3).

### 3.5. Cut-Off Values for METS-VF to Predict the Presence of CAC, Mild CAC, Moderate CAC, and Severe CAC

The sensitivity, specificity, Youden index and the optimal cut-off values of METS-VF to predict the severity of CAC are listed in Table 4. The optimal cut-off value of METS-VF was 6.405 for predicting the presence of CAC, 6.405 for mild CAC, 6.517 for moderate CAC, and 6.532 for severe CAC, respectively.

## 4. Discussion

The present study investigates the association between METS-VF and CAC, with CACS serving as a robust indicator of atherosclerosis and cardiovascular risk. Notably, METS-VF, a novel tool for estimating visceral fat, emerges as an independent predictor capable of detecting both the presence and severity of coronary calcification. CVD remains a leading cause of global mortality, highlighting the importance of accurate risk stratification in asymptomatic individuals. Among the various anthropometric indices examined, METS-VF consistently outperforms in predicting both the presence and extent of CAC. By exploring the relationship between METS-VF and CAC, and comparing it with other major anthropometric indices, our study emphasizes the potential value of METS-VF in enhancing cardiovascular risk assessment.

The correlation between anthropometric indices and CAC is well-documented, highlighting the association between obesity and an elevated risk of CAC [16]. While BMI, the initial obesity assessment tool, lacks precision in revealing body composition, WC proves more effective in predicting cardiovascular risk, serving as a direct measure of visceral fat [26]. VAI, integrating BMI and WC with TG and HDL cholesterol, correlates with obesity and is linked to CVD and all-cause mortality [27]. TyG index and METS-IR, incorporating fasting sugar and TG, display associations with CVD, with METS-IR showing potential superiority in predicting CAC compared to other insulin resistance indices like TyG index and TG/HDL cholesterol ratio [19,28]. METS-VF, derived from METS-IR, serves as a validated estimator of visceral fat, effectively predicting incident type 2 diabetes and hypertension; its enhanced predictive accuracy for CVD, enriched by age, gender, and WC components [12]. This study highlights METS-VF as a promising predictor for CAC, showcasing its superiority over other anthropometric indices, including BMI, WC, VAI, and the TyG index.

Our study has uncovered that METS-VF functions as an independent predictor, encompassing not only the presence of CAC but also various degrees of coronary calcification, spanning mild, moderate, and severe CAC. The severity of the CAC score holds the capability to predict the risk linked to mortality associated with all-cause, CVD, coronary heart disease, and even cancer [29,30]. In the context of clinical practice, the CACS emerges as a conceivable tool for risk assessment among asymptomatic patients, aiding in the formulation of primary prevention strategies such as statins and aspirin in line with the 2019 American College of Cardiology/American Heart Association Guideline on the Primary Prevention of CVD [31] and the 2021 European Society of Cardiology Guidelines on CVD prevention [32]. The obstacle to widespread CAC testing for screening lies in both radiation exposure concerns and the absence of routine coverage by insurance companies. Considering the increased costs and necessary exposure to radiation associated with CACS measurement, METS-VF could emerge as a promising tool for early cardiovascular risk identification in asymptomatic individuals.

Our study demonstrated that the optimal cut-off for METS-VF in predicting CACS > 0 is 6.405, and, as the severity of coronary calcification increases, the cut-off value for METS-VF also rises, reaching 6.532 in the prediction of severe CAC. A recent work reported that the optimal predictive values for visceral adiposity are 6.4 in men and 6.5 in women [33]. Meanwhile, a study in the Mexican population indicated that using the METS-VF > 7.18 cut-off value performs best in predicting visceral fat and the likelihood of developing type 2 diabetes and hypertension [12]. Another research study in the Chinese population showed that the cut-off value of METS-VF was 6.89 in men and 6.74 in women for predicting chronic kidney disease [15]. The cut-off values for METS-VF in our study are slightly lower than those reported in the Mexican and Chinese populations; nevertheless, the prediction outcomes using METS-VF differ from those observed in the present study. Notably, the METS-VF cut-off values in our study align with the work by Torun et al. [33], supporting the view that the METS-VF accurately reflects the visceral fat in association with CAC. This not only underscores the association between METS-VF and cardiovascular risk but also emphasizes the need to proactively address the increase in METS-VF.

There are several possible mechanisms to explain the association between visceral fat and CVD. First, adipose tissue consists of multiple cell types, including adipocytes, monocytes/macrophages, pericytes, endothelial cells, and various stem cells. Healthy adipose tissue is crucial for maintaining metabolic homeostasis, whereas dysfunctional adipose tissue significantly contributes to the development of metabolic diseases [34]. Dysfunctional adipose tissue secretes adipocytokines with pro-inflammatory and pro-oxidant effects [35]. In patients with type 2 diabetes, an increased level of resistin was linked to cerebrovascular symptomatology. Additionally, leptin demonstrated a positive correlation with the development of lipid cores and inflammatory cell infiltration within carotid plaques, while the adipokine chemerin displayed a negative association with carotid plaque stability [34]. Adipose tissue inflammation caused by obesity is an important component of insulin resistance, leading to type 2 diabetes and CVD [36]. Furthermore, perivascular adipose tissue (PVAT), surrounding the vascular wall, is a key regulator of vascular biology. As individuals experience obesity, the cellular composition and signaling pathways of PVAT shift in a way that promotes cardiovascular pathogenesis by increasing the generation of reactive oxygen species and inflammation [34]. Finally, visceral fat expansion derived from obesity may directly compress the kidney thereby activate the sympathetic nerve system and renin–angiotensin–aldosterone system to result in hypertension [37]. Enhancing our comprehension of adipose biology can potentially facilitate the development of enhanced therapies for CVD.

Dysfunctional adipose tissue, particularly in obesity, leads to an imbalance in adipokines production, which exacerbates vascular calcification by promoting osteogenic differentiation of vascular smooth muscle cells (VSMCs) and triggering vascular inflammation [38]. Adiponectin plays a protective role in preventing vascular calcification through several mechanisms. It inhibits the osteogenic differentiation of VSMCs by down-regulating the expression of Runx2, a key transcription factor in bone formation, and reducing the formation of extracellular matrix mineral nodules [39]. Additionally, adiponectin activates the AMP-activated protein kinase pathway, enhancing the Gas6/PI3K/AKT signaling and reducing apoptosis in VSMCs, which otherwise contribute to calcification [40]. Studies in adiponectin-deficient mice have shown spontaneous arterial calcification, further demonstrating adiponectin’s role in suppressing this process [39]. Bello-Chavolla et al. found a negative correlation between METS-VF values and adiponectin, with individuals having higher METS-VF displaying significantly lower plasma adiponectin levels compared to those with lower METS-VF [12]. These findings may help explain the link between visceral fat, as assessed by METS-VF, and vascular calcification.

However, our study had several limitations. Firstly, being a cross-sectional study, it cannot establish a causal relationship between METS-VF and CAC. Future longitudinal studies are needed to clarify the causal relationship of this association and to explore the link between CAC progression, cardiovascular outcomes, and changes in METS-VF over time. Secondly, the study population was limited to individuals receiving health examinations at a single hospital, potentially limiting the generalizability of the findings. Including more diverse and representative samples would enhance the external validity of our results. Expanding future studies to multiple regions and countries would further validate the applicability of our findings across different populations. Lastly, other confounding factors such as dietary habits, physical activity, and genetic background were not considered in the present study. These factors are known to influence both visceral fat and cardiovascular health and should be incorporated into future studies. To enhance the accuracy and robustness of our findings, further research should aim to include a comprehensive set of variables to better account for potential confounding effects.

In addition to addressing these limitations, future research should explore the development of therapeutic interventions targeting visceral fat reduction and adipokines to mitigate vascular calcification. Furthermore, integrating METS-VF into improved diagnostic algorithms or existing healthcare systems, possibly incorporating advanced imaging techniques and biomarkers, could enhance early detection and risk stratification for CVD related to visceral fat accumulation.

## 5. Conclusions

We found that METS-VF is an independent predictor of CAC presence and severity. Compared to traditional measures such as BMI, WC, VAI, TyG index, and METS-IR, METS-VF demonstrated a significantly stronger ability to distinguish CAC presence and different categories of CAC severity. This highlights the potential clinical utility of METS-VF as a valuable tool for cardiovascular risk assessment. For the future direction of research, investigating the underlying mechanisms linking METS-VF, as well as exploring the potential role of METS-VF in risk stratification and preventive strategies for CVD across different ethnicities and health systems, can be considered.

## Figures and Tables

**Table 1:** Baseline characteristics of study participants stratified by the tertiles of METS-VF.

Variables	All Participants (*n* = 1217)	Lowest METS-VF Tertile (*n* = 405)	Middle METS-VF Tertile (*n* = 406)	Highest METS-VF Tertile (*n* = 406)	*p*-Value
Age (years)	50.7 ± 9.9	47.4 ± 9.7	50.8 ± 9.1	53.9 ± 9.9	<0.001
Male, *n* (%)	655 (53.8)	89 (22.0)	231 (56.9)	335 (82.5)	<0.001
Hypertension, *n* (%)	234 (19.2)	20 (4.9)	71 (17.5)	143 (35.2)	<0.001
Diabetes, *n* (%)	58 (4.8)	3 (0.7)	15 (3.7)	40 (9.9)	<0.001
Current smoking, *n* (%)	82 (6.7)	9 (2.2)	22 (5.4)	51 (12.6)	<0.001
Systolic BP (mmHg)	124.7 ± 15.6	117.3 ± 13.9	125.7 ± 15.3	131.0 ± 14.3	<0.001
Diastolic BP (mmHg)	76.3 ± 11.7	71.2 ± 10.3	76.7 ± 11.3	81.0 ± 11.4	<0.001
BMI (kg/m^2^)	25.1 ± 4.0	21.6 ± 2.2	24.8 ± 2.2	28.8 ± 3.6	<0.001
WC (cm)	82.4 ± 10.9	71.3 ± 5.5	82.3 ± 4.6	93.4 ± 7.7	<0.001
VAI	1.7 ± 1.6	1.1 ± 0.9	1.7 ± 1.5	2.2 ± 2.0	<0.001
TyG index	8.5 ± 0.6	8.2 ± 0.5	8.6 ± 0.6	8.9 ± 0.6	<0.001
METS-IR	37.0 ± 8.4	29.5 ± 4.2	36.5 ± 4.8	45.0 ± 7.1	<0.001
METS-VF	6.2 ± 0.8	5.4 ± 0.5	6.3 ± 0.2	7.0 ± 0.3	<0.001
Fasting glucose (mg/dL)	100.6 ± 18.9	94.2 ± 9.0	99.8 ± 17.0	107.9 ± 20.7	<0.001
Total cholesterol (mg/dL)	207.3 ± 39.2	210.9 ± 39.2	209.5 ± 36.3	201.6 ± 41.2	0.001
HDL cholesterol (mg/dL)	53.5 ± 14.6	62.8 ± 14.9	52.4 ± 12.2	45.4 ± 10.9	<0.001
LDL cholesterol (mg/dL)	125.5 ± 34.7	124.1 ± 34.7	128.1 ± 32.4	124.3 ± 36.7	0.153
Triglyceride (mg/dL)	102.0 (67.5–149.0)	72.0 (53.0–102.5)	105.0 (74.0–155.0)	130.5 (95.0–175.3)	<0.001
Uric acid (mg/dL)	5.9 ± 1.4	5.1 ± 1.1	6.1 ± 1.3	6.5 ± 1.4	<0.001
Serum creatinine (mg/dL)	0.83 ± 0.20	0.72 ± 0.17	0.84 ± 0.20	0.91 ± 0.19	<0.001
eGFR (mL/min/1.73 m^2^)	95.4 ± 14.6	101.4 ± 13.3	94.5 ± 13.4	90.2 ± 14.9	<0.001
CACS > 0, *n* (%)	375 (30.8)	62 (15.3)	111 (27.3)	202 (49.8)	<0.001
Mild CAC	258 (68.8)	50 (80.6)	83 (74.8)	125 (61.9)	<0.001
Moderate CAC	79 (21.1)	9 (14.5)	20 (18.0)	50 (24.8)	<0.001
Severe CAC	38 (10.1)	3 (4.8)	8 (7.2)	27 (13.4)	<0.001

Abbreviations: METS-VF, metabolic score for visceral fat; BP, blood pressure; BMI, body mass index; WC, waist circumference; VAI, visceral adiposity index; TyG, triglyceride–glucose; METS-IR, metabolic score for insulin resistance; HDL, high-density lipoprotein; LDL, low-density lipoprotein; eGFR, estimated glomerular filtration rate; CACS, coronary artery calcium score; CAC, coronary artery calcification.

**Table 2:** Correlation of METS-VF and anthropometric indices with CACS.

Variables	Spearman’s ρ	*p*-Value
BMI	0.182	<0.001
WC	0.243	<0.001
VAI	0.110	<0.001
TyG index	0.180	<0.001
METS-IR	0.220	<0.001
METS-VF	0.349	<0.001

**Table 3:** Associations of METS-VF and anthropometric indices with mild, moderate, and severe CAC.

	Unadjusted OR (95% CI)			Adjusted OR (95% CI)		
	Mild CAC (CACS 1–99)	Moderate CAC (CACS 100–399)	Severe CAC (CACS ≥ 400)	Mild CAC (CACS 1–99)	Moderate CAC (CACS 100–399)	Severe CAC (CACS ≥ 400)
BMI (per 1-SD)	1.378 (1.205–1.577) *	1.398 (1.125–1.739) *	1.741 (1.302–2.328) *	1.137 (0.947–1.366)	1.049 (0.734–1.499)	1.487 (0.888–2.490)
WC (per 1-SD)	1.607 (1.391–1.956) *	1.793 (1.411–2.277) *	2.362 (1.690–3.303) *	1.176 (0.949–1.456)	1.171 (0.789–1.738)	1.493 (0.852–2.616)
VAI (per 1-SD)	1.203 (1.057–1.369) *	1.319 (1.095–1.589) *	1.041 (0.740–1.463)	0.911 (0.506–1.640)	0.559 (0.213–1.473)	0.333 (0.047–2.370)
TyG index (per 1-SD)	1.426 (1.241–1.638) *	1.763 (1.417–2.194) *	1.428 (1.042–1.956) *	1.046 (0.752–1.455)	2.069 (1.122–3.815) *	1.989 (0.709–5.583)
METS-IR (per 1-SD)	1.494 (1.301–1.717) *	1.633 (1.313–2.031) *	1.804 (1.342–2.425) *	1.220 (0.957–1.555)	1.212 (0.754–1.949)	1.745 (0.858–3.548)
METS-VF (per 1-SD)	2.259 (1.872–2.727) *	3.460 (2.406–4.974) *	5.913 (3.309–10.564) *	1.450 (1.115–1.886) *	1.865 (1.137–3.062) *	2.316 (1.090–4.923) *

* *p*-value < 0.05. Abbreviations: OR, odds ratio; CI, confidence interval; SD, standard deviation; other abbreviations are as listed in Table 1. Covariates in the multivariable model included age, sex, hypertension, diabetes, current smoking, total cholesterol, HDL cholesterol, LDL cholesterol, triglyceride, uric acid, and eGFR.

**Table 4:** Sensitivity, specificity, and Youden index using cut-off values for METS-VF to predict the severity of CAC.

	Cut-Off Value	Sensitivity (%)	Specificity (%)	Youden Index
Presence of CAC	6.405	69.9	63.0	0.328
Mild CAC	6.405	65.9	62.9	0.288
Moderate CAC	6.517	77.2	68.5	0.457
Severe CAC	6.532	78.9	69.6	0.485

## Data Availability

The data that support the findings of this study are available from the corresponding author on reasonable request.

## Supplementary Materials

The following supporting information can be downloaded at: https://www.mdpi.com/article/10.3390/life14111399/s1: Figure S1: Relationship between the severity of coronary artery calcification (CAC) and METS-VF; Table S1: Comparisons of the differences in AUC for prediction of different severity of CAC between METS-VF and anthropometric indices using DeLong’s test; Table S2: Integrated discrimination improvement and net reclassification improvement by adding METS-VF to the model including covariables in predicting the presence of CAC; Table S3: Integrated discrimination improvement and net reclassification improvement by adding METS-VF or anthropometric indices to the model including covariables in predicting the presence of CAC.
