# Supplementary material for: Exploring the Relationship Between Visceral Fat and Coronary Artery Calcification Risk Using Metabolic Score for Visceral Fat (METS-VF)
Source: Life (Basel). 2024 Oct 31;14(11):1399. doi: 10.3390/life14111399 (PMC11595436; doi:10.3390/life14111399)
Supplement: Supplementary file 1 [file life-14-01399-s001.zip › life-3239459-supplementary.pdf]

Table S1. Comparisons of the differences in AUC for prediction of different severity of CAC between METS-VF and anthropometric indices using DeLong's test

	**Presence of CAC (CACS >0)**		**Mild CAC (CACS 1−99)**		**Moderate CAC (CACS 100−399)**		**Severe CAC (CACS ≥400)**	
	AUC (95% CI)	*P*-value	AUC (95% CI)	*P*-value	AUC (95% CI)	*P*-value	AUC (95% CI)	*P*-value
METS-VF	0.710 (0.679−0.741)	Reference	0.682 (0.645−0.718)	Reference	0.757 (0.702−0.812)	Reference	0.807 (0.744−0.870)	Reference
METS-IR	0.634 (0.601−0.667)	<0.001	0.619 (0.580−0.657)	<0.001	0.654 (0.593−0.715)	<0.001	0.696 (0.628−0.765)	<0.001
BMI	0.610 (0.576−0.643)	<0.001	0.597 (0.558−0.635)	<0.001	0.607 (0.545−0.669)	<0.001	0.703 (0.634−0.772)	<0.001
WC	0.647 (0.615−0.680)	<0.001	0.628 (0.591−0.666)	<0.001	0.667 (0.609−0.726)	<0.001	0.736 (0.665−0.806)	<0.001
VAI	0.571 (0.536-0.606)	<0.001	0.567 (0.527-0.607)	<0.001	0.595 (0.522-0.668)	<0.001	0.551 (0.462-0.640)	<0.001
TyG index	0.611 (0.577−0.625)	<0.001	0.598 (0.560−0.637)	<0.001	0.654 (0.587−0.722)	0.007	0.609 (0.523−0.695)	<0.001

Abbreviations: AUC, area under curve; CAC, coronary artery calcification; CACS, coronary artery calcium score; METS-VF, metabolic score for visceral fat; METS-IR, metabolic score for insulin resistance; BMI, body mass index; WC, waist circumference; VAI, visceral adiposity index; TyG, triglyceride-glucose.

Table S2. Integrated discrimination improvement and net reclassification improvement by adding METS-VF to the model including covariables in predicting the presence of CAC

	**Integrated discrimination improvement**			**Net reclassification improvement**		
	IDI (%)	CI (%)	*P*-value	NRI (%)	CI (%)	*P*-value
Baseline model + METS-VF	0.83	0.31−1.35	0.002	25.1	13.1−37.2	<0.001

Baseline model was adjusted for age, sex, hypertension, diabetes, current smoking, total cholesterol, HDL cholesterol, LDL cholesterol, triglyceride, uric acid, and eGFR.

All estimates are provided with 95% confidence intervals.

Table S3. Integrated discrimination improvement and net reclassification improvement by adding METS-VF or anthropometric indices to the model including covariables in predicting the presence of CAC

Baseline model plus	Integrated discrimination improvement			Net reclassification improvement		
	IDI (%)	CI (%)	*P*-value	NRI (%)	CI (%)	*P*-value
METS-VF *vs*. BMI	0.68	0.31−1.06	<0.001	17.7	5.6−29.8	0.004
METS-VF *vs*. WC	0.63	0.31−0.96	<0.001	20.7	8.6−32.7	<0.001
METS-VF *vs*. VAI	0.81	0.28−1.34	0.003	23.5	11.4−35.5	<0.001
METS-VF *vs*. TyG index	0.77	0.25−1.29	0.004	19.9	7.8−32.0	0.001
METS-VF *vs*. METS-IR	0.58	0.22−0.95	0.002	13.3	1.1−25.4	0.033

Baseline model was adjusted for age, sex, hypertension, diabetes, current smoking, total cholesterol, HDL cholesterol, LDL cholesterol, triglyceride, uric acid, and eGFR.

All estimates are provided with 95% confidence intervals.

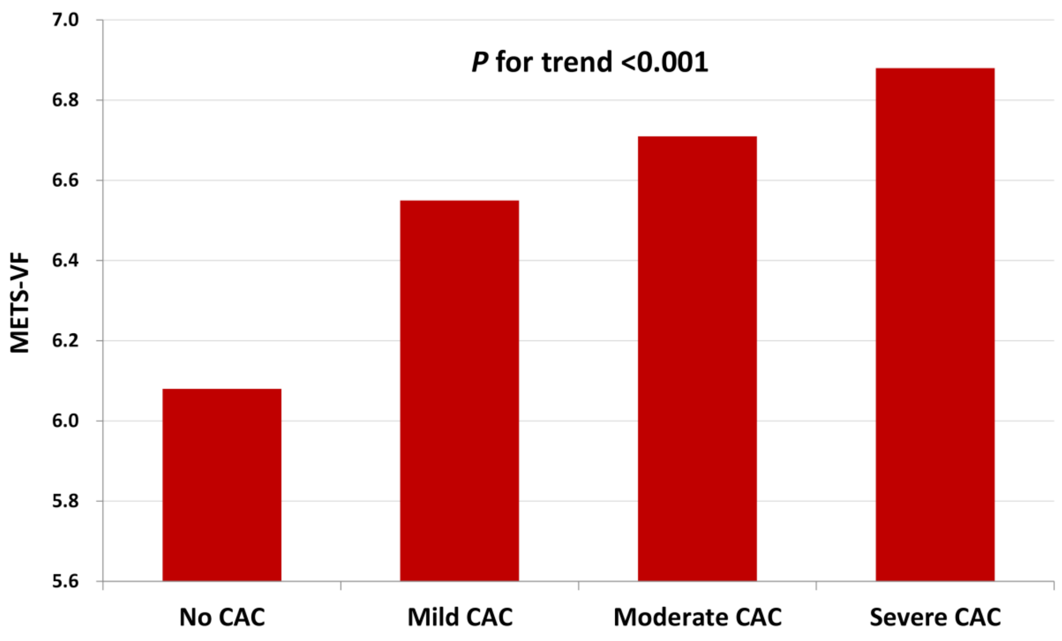

Figure S1. Relationship between the severity of coronary artery calcification (CAC) and METS-VF
